# Food Shopping and Acquisition Behaviors in Relation to BMI among Residents of Low-Income Communities in South Carolina

**DOI:** 10.3390/ijerph14091075

**Published:** 2017-09-16

**Authors:** Angela D. Liese, Xiaonan Ma, Brent Hutto, Patricia A. Sharpe, Bethany A. Bell, Sara Wilcox

**Affiliations:** 1 Department of Epidemiology and Biostatistics, Arnold School of Public Health, University of South Carolina, 915 Greene Street, Columbia, SC 29208, USA; xma@email.sc.edu; 2 Prevention Research Center, Arnold School of Public Health, University of South Carolina, 921 Assembly Street, Columbia, SC 29208, USA; brent@brenthutto.com (B.H.); sharpep@mailbox.sc.edu (P.A.S.); wilcoxs@mailbox.sc.edu (S.W.); 3 College of Social Work, University of South Carolina, 1512 Pendleton Street, Columbia, SC 29208, USA; bellb@mailbox.sc.edu; 4 Department of Exercise Science, Arnold School of Public Health, University of South Carolina, 921 Assembly Street, Columbia, SC 29208, USA

**Keywords:** shopping distance, shopping frequency, grocery store type, community food resources, food assistance, food security

## Abstract

Low-income areas in which residents have poor access to healthy foods have been referred to as “food deserts.” It is thought that improving food access may help curb the obesity epidemic. Little is known about where residents of food deserts shop and if shopping habits are associated with body mass index (BMI). We evaluated the association of food shopping and acquisition (e.g., obtaining food from church, food pantries, etc.) with BMI among 459 residents of low-income communities from two South Carolina counties, 81% of whom lived in United States Department of Agriculture-designated food deserts. Participants were interviewed about food shopping and acquisition and perceptions of their food environment, and weight and height were measured. Distances to food retail outlets were determined. Multivariable linear regression analysis was employed. Our study sample comprising largely African-American women had an average BMI of 32.5 kg/m^2^. The vast majority of study participants shopped at supermarkets (61%) or supercenters/warehouse clubs (27%). Shopping at a supercenter or warehouse club as one’s primary store was significantly associated with a 2.6 kg/m^2^ higher BMI compared to shopping at a supermarket, independent of demographics, socioeconomics, physical activity, and all other food shopping/acquisition behaviors. Persons who reported shopping at a small grocery store or a convenience or dollar store as their tertiary store had a 2.6 kg/m^2^ lower BMI. Respondents who perceived lack of access to adequate food shopping in their neighborhoods as a problem had higher BMI. Living in a food desert census tract was not significantly associated with BMI. Other shopping attributes, including distance to utilized and nearest grocery stores, were not independently associated with BMI. These findings call into question the idea that poor spatial access to grocery stores is a key underlying factor affecting the obesity epidemic. Future research should consider assessing foods purchased and dietary intake within a comprehensive study of food shopping behaviors and health outcomes among persons living in food deserts.

## 1. Introduction

In the past decade, a number of studies have examined whether residents of socioeconomically disadvantaged neighborhoods are more likely to be obese than those of more affluent neighborhoods [1,2,3,4,5]. These studies have relied on geographic information systems (GIS) to characterize neighborhood characteristics combined with information on residents’ health status. This approach was further expanded to include characterization of the neighborhoods with respect to food availability and accessibility characteristics [6,7], which ultimately led to the landmark report by the United States (US) Department of Agriculture’s Economic Research Service on “Access to Affordable and Nutritious Food: Measuring and Understanding Food Deserts and Their Consequences” [8] and the coining of the term “food deserts” [9].

A key assumption of this line of research has been that people actually shop in the neighborhoods in which they live. However, several studies have now shown this is not the case, as many shoppers do not select the nearest grocery store [10,11,12,13,14,15,16]. For example, in a study of residents of the South Carolina Midlands region, we found that the utilized grocery stores were on average 3.2 miles farther away than the nearest stores for residents of food deserts and 4.2 miles farther away for residents of non-deserts [10]. It is therefore not surprising that, in that same population, the characteristics of the built environment (e.g., availability of stores in the neighborhood) were not directly associated with fruit and vegetable intake (they were indirectly associated) and that, instead, actual food shopping behaviors (e.g., frequency of grocery shopping) were the central characteristics associated with fruit and vegetable intake [17]. Another hypothesis tested by previous research has been that shopping at supermarkets or large grocery stores is associated with healthier grocery selection, healthier eating behavior, and lower body mass index (BMI) [18], and some studies have indeed shown store type to be associated with quality of dietary intake [15,16,18,19,20,21]; however, no studies focusing on BMI have considered the actual types of foods being purchased.

Very few studies have collected detailed information on food shopping behaviors, including characteristics of the stores utilized (e.g., type, price level, distance, etc.) and mode of transportation, and have been able to relate these to obesity [11,12,15,18,21,22,23,24,25,26]. Although findings have been mixed with respect to which food shopping behaviors are associated with obesity, there seems to be some consistency, with studies finding few associations between obesity and distance to utilized store [11,12,15,22] but relatively consistent evidence for certain store types—particularly discount grocery stores—being associated with higher BMI and obesity [11,12,21,24]. However, most studies to date have focused their assessment of food shopping behaviors on the primary store, even though most households tend to utilize multiple stores [25,27].

Thus, the purpose of this analysis was to comprehensively evaluate the association of food shopping at the three most frequented grocery stores and food acquisition behaviors (e.g., obtaining food at churches or social service organizations, food banks or pantries) with BMI among residents of two low-income communities in South Carolina, 81% of whom lived in food desert census tracts, defined as a tract with a low-income population having low access to a supermarket or supercenter [8,28,29]. We hypothesized that distance to and type of grocery store utilized; frequency of shopping; acquiring food at food banks or pantries or from a church or other social service organization; perceptions of the built food environment; and food environment access characteristics would be significant predictors of BMI. These hypotheses were based on a conceptual framework we developed previously [17].

## 2. Materials and Methods

### 2.1. Study Design

This cross-sectional study is a secondary analysis using baseline data from an evaluation study of a healthy food access initiative, described previously [27,30]. In brief, the study was funded to evaluate the impact of a food hub intervention to increase healthy food access with a longitudinal, quasi-experimental design among a low-income population. The target population of this study comprised: (a) residents of high-poverty areas (i.e., a poverty level greater than or equal to the state’s level of 16%) of the food hub’s service area (Location 1); and (b) residents of areas with similar poverty levels in another part of the state in which no food hub was present or planned (Location 2). Recruitment focused on seven census tracts (six of which were USDA-designated urban food deserts [8,28,29] in two distinct locations (i.e., four tracts in Location 1 and three tracts in Location 2) in South Carolina that were selected to have similar demographic, socioeconomic, and health-related characteristics. To accommodate local community definitions of neighborhoods, which extend beyond the geographic boundaries of census tracts, we extended the eligibility boundaries for participant recruitment to 1 mile past the seven recruitment tract boundaries into adjacent tracts, but only if the adjacent tract had a poverty level greater than or equal to that of the state (≥16% of households below the federal poverty level). This yielded an expanded participant recruitment area that included residents of 19 tracts, of which 12 were food deserts. Thus, the majority (i.e., 89% of participants in Location 1 and 74% in Location 2) resided in food desert census tracts. Baseline data were collected between November 2013 and May 2014.

The targeted enrollment goal of 560 individuals for the evaluation study was determined based on pre-specified hypotheses, statistical power analysis, and assumptions about retention rates over time. Using purchased address lists from a survey sampling firm, letters addressed to the “family food shopper” were mailed to all residential addresses in the seven tracts where recruitment was focused (*n* = 6136) inviting him/her to call for information about a study of food access and food shopping. Multiple recruitment strategies (in-person, printed, and electronic) followed this initial letter. Eligibility screening occurred by phone or in-person at community locations. The in-person interview, conducted with 527 participants prior to the food hub’s opening, included sociodemographic, attitudinal, behavioral, food shopping, and health-related questions. The study was reviewed and approved by the Institutional Review Board of the University of South Carolina (approval number Pro00023844).

### 2.2. Assessment of Food Shopping and Acquisition

Participants were interviewed with respect to their food shopping behaviors in retail stores, as well as acquisition of foods in other settings, some of which offer food free of charge. Participants were prompted to think about the past year and where they did their usual shopping and were then asked: “What is the name of the store or market where you shopped the most for food (Store 1)”, “What is the name of the store where you shopped the second most often (Store 2)?”, and “…the third most often (Store 3)?” Subsequently, information was collected on the addresses of stores 1 to 3, the type of store (convenience stop, drugstore/pharmacy, dollar variety store, farmers market, food bank or food pantry (which was never affirmed), supermarket, supercenter, smaller grocery store, specialty store, warehouse club, or other type of food store, such as a military commissary), and the shopping frequency at each store (i.e., for Store 1: “Over the past year, how often did you usually shop at (name of primary store answered before)?”). Respondents could answer in their preferred unit of time as they responded to shopping frequency. Because shopping frequency varied widely across the three stores, we applied store-specific cutpoints (i.e., Store 1: <1 time per week vs. ≥1 time per week; Store 2: <2 times per month vs. ≥2 times per month; Store 3: <1 time per month vs. ≥1 time per month). Greater grocery shopping frequency has been associated with greater fruit and vegetable intake, and thus the more frequent category was made the referent group [17]. Store type was grouped into supermarkets (referent group), supercenters/warehouse clubs, and all other store types.

Transportation mode to Store 1 was queried in six categories (i.e., drive their own car/van/truck/motorcycle; ride in a car/van/truck/motorcycle of family or a friend; ride a bus; take a taxi; walk; ride a bicycle) and was reclassified into four categories by combining riding a bus with riding in a taxi, and walking with riding a bicycle. Participants also reported whether they shopped at a farmers’ market or whether they acquired food from a food bank or food pantry or from a church or social service organization.

### 2.3. Assessment of Perceptions of the Food Environment

Perceptions of the food environment were assessed using the questions previously developed for the Multi-Ethnic Study of Atherosclerosis (MESA) Neighborhood Study [31,32,33]. Respondents were asked to think of their neighborhood as the area within a 20-min walk or 1 mile (1.6 km) from home [31,32] and were asked if they agreed or disagreed with the following statements: (1) “A large selection of fresh fruits and vegetables is available in my neighborhood.”; (2) “The fresh fruits and vegetables in my neighborhood are of high quality.”; (3) “A large selection of low-fat products is available in my neighborhood.”; and (4) “There are many opportunities to purchase fast foods in my neighborhood.” Participants were also asked: (5) “How much of a problem would you say that lack of access to adequate food shopping is in your neighborhood?” Participants responded on a Likert scale ranging from 1 (strongly agree) to 5 (strongly disagree), except for question 5, for which the responses ranged from 1 (very serious problem) to 4 (not really a problem). Because we wished to have consistent interpretation of higher values of perception variables as indicative of healthier/better food access, the responses were reverse-coded for questions 1, 2, and 3. In line with previous research [31], we also created a composite healthy food availability scale as the sum of questions 1, 2, and 3. For analyses, all perception scales were transformed to have a minimum value of zero.

### 2.4. Assessment of Demographic and Socioeconomic Characteristics, Physical Activity, Height, and Weight

Participants were asked their age, self-identified race, gender, marital status, and the number of adults and children in the household for whom they shopped. Participants were additionally asked whether they or anyone in their household received any benefits from either of the two largest food and nutrition assistance programs in the US, the Supplemental Nutrition Assistance Program (SNAP) or the Women, Infants and Children (WIC) food and nutrition assistance program. Annual total household income was queried and recorded in six categories of $10,000 and subsequently grouped into three categories (i.e., <$10,000, $10,000–19,000, and ≥$20,000) based on the distribution. Highest level of education completed was reported as a categorical variable in seven levels and recoded as less than high school, high school (which included GED and high school diploma), and some college and above. Food security was assessed by interview using the validated 18-item USDA US-Household Food Security Survey Module (HFSSM) [34]. Food security was classified into three categories (i.e., high and marginal food security (1–2 affirmative responses on HFSSM), low food security (3 to 7 affirmative responses), and very low food security (≥8 affirmative responses)). Physical activity was queried as the number of days in which a person engaged in physical activity that increased his/her breathing rate for 30 min or more [35]. Trained staff measured weight in pounds using a Seca electronic scale calibrated per the manufacturer’s instructions and height with a Seca stadiometer on a stable surface. Height was measured to the nearest half centimeter, and weight was measured to the nearest 1/10 pound. Pounds were converted to kilograms, and BMI was computed as weight in kg/height in meters^2^.

### 2.5. GIS Analyses and Ground-Truthing of the Built Food Environment

First, the participants’ residential addresses were geocoded using ArcGIS 10.2 (Environmental Systems Research Institute, Inc, Redlands, CA, USA) and 2013 Topologically Integrated Geographic Encoding and Referencing (TIGER) road files for 2013 [36]. Subsequently, the geographic area for the ground-truthing survey was generated by overlaying: (1) the original seven census tracts used for participant recruitment; with (2) the 1-mile external Euclidian buffer around the tracts (without consideration of the poverty criteria); and (3) a 1-mile external Euclidean buffer around each participant’s home address. These areas were then merged into one contiguous study area for each of the two study locations. In total, 35 whole or partial census tracts were included, of which 15 were food deserts.

The purpose of the ground-truthing survey was to verify the location of all food retail outlets in these areas and obtain Global Positioning System (GPS) coordinates. Local field staff canvassed the areas and recorded the GPS coordinates, name, type, and address of each food retail outlet using a Garmin GPSMAP 62 receiver (Garmin, Olathe, KS, USA). The information from the ground-truthing survey was used to calculate measures of food environment access and distance to the nearest stores and characterize the participants’ food environment.

Lastly, two data sources were developed and integrated to calculate network distance measures to utilized food retail outlets in ArcGIS 10.2. For participants whose utilized grocery stores fell within the ground-truthed area, we computed the network distance from their residence to the GPS location of the verified store. Additionally, for study participants who shopped outside the ground-truthed areas, their lists of utilized stores (outside the ground-truthed area) were appended with geocoordinate data for those specific stores extracted from the South Carolina Department of Health and Environmental Control (SC DHEC) food retail licensing database from 2012 to 2013, which includes geocodes of all food retail outlets. According to a previous validation, the SC DHEC database is one of the best secondary food outlet datasets, with a sensitivity (the fraction of open food outlets that were listed and found to be open during the field census) of 68% and a positive predictive value (the fraction of all listed food outlets that were located and open during the field census) of 89% [37].

### 2.6. Statistical Analysis

Of the 527 participants, a total of 68 were excluded for the following reasons, leaving a total of 459 participants for analyses: pregnant (*n* = 7), missing height/weight measurements (*n* = 5), missing demographic information (*n* = 3), missing income information (*n* = 13), missing information or did not indicate a store for store 1, 2, or 3 (*n* = 5), missing transportation information to store 1 (*n* = 33), and missing responses to perceptions of the food environment questions (*n* = 2). An assessment of the missing data mechanism revealed no systematic patterns in the small amount of missing data. Descriptive analyses of the sample characteristics were performed using SAS software (version 9.4, SAS Institute Inc., Cary, NC, USA) [38]. Because there were a few extreme distance values, all distances were Winsorized at the 95th percentile [39].

Hierarchical linear regression was used to quantify associations between BMI and several sequential sets of predictors, as informed by our previous work and the conceptual framework developed therein [17]. The three models estimated contained sets of independent variables as follows, all adjusted for demographic and socioeconomic covariates and for physical activity: Model 1 focused on food shopping characteristics (i.e., distance to utilized stores, frequency of shopping at utilized stores, type of stores utilized, etc.) and utilization of community food resources; Model 2 added perceptions of the food environment to Model 1; and Model 3 added built food environment characteristics to Model 2, including food desert status of the census tract of residence. Tolerance values were examined to check for multicollinearity among the predictors, and the distributional assumptions (normality, linearity, and homoscedasticity of the residuals) were also examined. With tolerance values ranging from 0.35 to 0.91, there was no indication of collinearity. The residual plots did not reveal any violations of assumptions. In addition to examining the overall model R^2^, we also calculated the uniqueness index (i.e., squared semi-partial correlation) for each predictor. All data management and analyses were conducted in SAS v9.4. All significance tests were conducted at the 0.05 level.

## 3. Results

Participants were approximately equally distributed across the two study locations in South Carolina (47% vs. 53%), and 89% of participants in Location 1 and 74% in Location 2 resided in food desert census tracts.

Characteristics of the study population with complete data for this analysis are summarized in Table 1. Most of the participants were African American (93%) and female (80%); had an average age of 52 years; and only about 13% were married or living with a partner. A large percentage were food insecure (62%), participated in SNAP (66%), had completed at most high school or a lower level of education (69%), and had an annual household income less than $20,000 (79%). The average BMI was 32.5 kg/m^2^, and 55% of the sample was obese.

Table 1 additionally shows food shopping characteristics of participants’ three most-frequented stores. On average, participants traveled 2.5 miles to their primarily utilized store and slightly farther to stores 2 and 3. Only 45% of the sample used a vehicle of their own to get to store 1; 36% rode in someone else’s vehicle, 8.9% took a bus or taxi, and 10% walked or used a bicycle. Supermarkets were the most frequented type of store utilized (61% for store 1, 64% for store 2, and 67% for store 3), followed by supercenters (27%, 22%, and 14%, respectively). Only a small percentage (i.e., 12%, 14%, and 19%, respectively) selected another store type (e.g., convenience, drug/pharmacy, dollar, specialty, or smaller grocery store) for stores 1, 2, and 3. Shopping frequency varied substantially across the utilized stores, with about 60% of participants shopping less than once a week at their primary store, 34% shopping less than twice a month at store 2, and 66% shopping less than once a month at store 3.

The majority of participants indicated that they additionally acquired food at food banks or food pantries (53%) or from a church or social service organization (54%), and less than half (46%) shopped at a farmers’ market in the previous year. The nearest supermarket/large grocery store was on average 1.5 miles away, and the nearest supercenter/warehouse club was 2.7 miles away.

Participants largely perceived their food environment as not supporting healthy food availability (average 4.7 on a scale ranging from 0 to 12), on average considered lack of access to adequate food shopping a minor or a somewhat serious problem (average 1.5 on a scale ranging from 0 to 3), and were relatively neutral in terms of having opportunities to purchase fast foods (average 1.8 on a scale ranging from 0 to 4).

Table 2 presents results from the hierarchical linear models. Shopping at a supercenter/warehouse club for store 1 compared to shopping at a supermarket was positively and significantly associated with BMI in Model 1, and shopping at an “other” type of store for store 3 was inversely associated with BMI, after controlling for demographic and socioeconomic covariates. Acquiring food at food banks/pantries or churches/social services or a farmers’ market was not associated with BMI. These findings remained unchanged when participant perceptions of the food environment were added to the model (Model 2). Expressing the perception that lack of access to adequate food shopping in the neighborhood was not really a problem was inversely associated with BMI, i.e., the less a person considered lack of access a problem, the lower his/her BMI. Perceptions of healthy food availability or fast food availability were not significantly associated with BMI. The correlation between the lack of access question (i.e., access not really being a problem) and the availability of healthy foods in the neighborhood score was significant but moderate in size (r = 0.37). Further consideration of the minimum distance a participant would have to travel to reach the nearest supermarket or supercenter/warehouse club and whether the participant resided in a food desert census tract (Model 3) did not change the findings. Food desert status of the census tract of residence was not significantly associated with BMI. Additionally, the model R^2^ increased across the models from 0.15754 in Model 1 to 0.1730 and 0.1768 in Models 2 and 3, respectively, with the increase in R^2^ being statistically significant. The small magnitude of the squared semi-partial correlations indicated that no one single predictor variable explained a substantial amount of the variation.

We additionally estimated the mean BMI levels for participants and differences between groups of participants from Model 3. Adjusted for all covariates, those shopping at supercenters/warehouse clubs for store 1 had a 2.6 kg/m^2^ higher BMI than those who shopped at supermarkets (31.6 kg/m^2^ vs. 28.9 kg/m^2^, respectively), and participants who shopped at an “other” type of store for store 3 had a 2.6 kg/m^2^ lower BMI than those who shopped at a supermarket for store 3 (27.3 kg/m^2^ vs. 29.9 kg/m^2^, respectively). In addition, a one-unit higher response on the lack of food access question (i.e., indicative of food access not being a problem) was associated with a 0.86 kg/m^2^ lower BMI.

## 4. Discussion

The present study focused on very low income populations in two Southern cities, the majority of whom were of minority race/ethnicity and resided in census tracts designated by the USDA as food desert tracts at the study’s inception. Ours was a population experiencing very high poverty, with 46% of shoppers reporting incomes below $10,000 and more than half acquiring food at food banks, food pantries, churches, and social service organizations. A similar population was recently studied in Pittsburg, Pennsylvania [15]. The majority of our study’s participants shopped at grocery stores inside the two geographic study areas, as defined by the ground-truthing survey (62–65% for stores 1–3). However, the utilized grocery stores within the study areas were all located in the non-food desert census tracts, where only 11% (in Location 1) and 26% (in Location 2) of the populations lived. Similar findings were reported by LeDoux and Vojnovic (2013), who demonstrated that residents of census tracts in the lower east side of Detroit, Michigan, generally did not utilize their immediate food environment for grocery shopping and selectively shopped elsewhere [14].

This study is one of a relatively small number that have focused explicitly on actual grocery shopping behaviors characterized by GIS measures to utilized stores and their relation to BMI. Consistent with several previous reports, we did not find evidence for an association of distance to the primarily utilized grocery store and BMI [11,12,15,22,26]. However, we additionally offer evidence that distance to the second or third most-frequented grocery store is also not associated with BMI. This suggests that previous null findings on distance and BMI in studies assessing only a single store are not likely to have been confounded by lack of information on other utilized stores, i.e., by potential trade-offs made by consumers with respect to time and distance to multiple utilized stores. Moreover, comparing the distance to each of the three stores suggested that participants chose a primary store that was on average slightly more proximal (0.2 miles) than their second most-frequented store, which was in turn 0.2 miles closer than the third store. All three stores were, on average, farther away than the nearest supermarket, which suggests that these shoppers do not in general shop at the most proximal location [10,11,12,13,14,15,16].

Contrary to our hypothesis, which had been informed by previous work [15,17,18], shopping frequency was not associated with BMI. Most studies to date have not included data on shopping frequency at utilized stores [11,12,22,23], and hence there are few data to which we can compare our findings. The average shopping frequency at the primary grocery store of 1.2 times per week found here is lower than the 1.8 times per week we reported in a different area of South Carolina [10]. In a study among SNAP recipients in eastern North Carolina, Jillcott-Pitts et al. (2012) demonstrated that the frequency of shopping at supercenters and supermarkets was associated with the healthfulness of foods purchased but, similar to our results, was not associated with BMI [18]. In a study situated in two mostly African-American neighborhoods with low-income residents in Pittsburgh, Pennsylvania, Dubowitz et al. (2015) did not find a relationship between shopping frequency and BMI or dietary intake quality [15].

An unexpected finding in our study was that using a supercenter (e.g., Walmart and Kmart) or a warehouse club (e.g., Sam’s Club) as the primary food store was associated with significantly higher BMI levels compared to shopping at a supermarket. Of note, 27% of study participants shopped at supercenters or warehouse clubs for store 1, 22% shopped at these stores for store 2, and 14% used them for store 3, with the vast majority of participants shopping at supercenters (i.e., only 1–3% shopped at warehouse clubs). Interestingly, Jilcotts-Pitts et al. (2012) and Minaker et al. (2016) did not see this distinction [18,21], and the initial positive association of supercenter use and BMI observed by Dubowitz et al. (2015) was attenuated after adjustment [15]. Drenowski et al. (2012) reported significantly higher rates of obesity among shoppers at medium- or low-price supermarkets than at higher-price supermarkets in a study in Seattle. Low-price supermarkets predominantly included FredMeyer and Albertson’s, and higher-price supermarkets included Puget Consumer Co-op and Whole Foods, among others [12]. Ghosh-Dastidar et al. found lower store prices in the utilized stores (but not distance to these stores) to be associated with higher obesity in the aforementioned Pittsburgh study [26]. In a study of residents of the Paris, France, region, Chaix et al. (2012) reported that shoppers at hard discounters (e.g., Aldi) with middle to lower levels of education had significantly higher BMI compared to those who did not shop at hard discounters [11]. Other studies of supercenter stores and BMI are more indirect: Gustafson et al. (2011) showed that individuals residing in a census tract containing a supercenter were heavier than individuals in a tract without a supercenter [40]. All of these findings raise challenging questions as to the inferences that can be made, which we discuss more below.

Additionally, we found that using a smaller store, i.e., a store in the “other” category (for example, a convenience store, drugstore, dollar store, small grocery store, or specialty store) as a third store was associated with significantly lower BMI compared to shopping at a supermarket. Minaker et al. (2016) comprehensively describe reasons for different store type choices in a study in Ontario, Canada [21]. They found that proximity and convenient hours were the most frequent reasons for choosing a convenience store, whereas quality and availability of specific foods were the key reasons for specialty store selections. Although they found no association of convenience store utilization and BMI, they found a significant inverse association of specialty store use and BMI. Given that we did not assess reasons for store choice, we can only hypothesize that choosing a smaller store as one’s third store may reflect a shopping behavior pattern that tailors selection of store type to the purpose of shopping trips and that the ability to be this selective may also indicate easier access to transportation.

A further interesting finding emerged from studying the association of a resident’s perceptions of his/her food environment with BMI, for which we utilized three different scores, one a composite score of the availability of healthful options in the neighborhood, one on availability of fast food outlets, and a more general question on whether access to food shopping was a problem. We found that the less a person considered lack of access a problem, the lower their BMI, or, conversely, the more a person considered lack of access a problem, the higher their BMI, and these findings were independent of the other food shopping behaviors and food environment characteristics, as well as the other two perception measures. The direction of these findings is consistent with both longitudinal and cross-sectional findings from MESA [41,42], which reported that residents that perceived their neighborhood as the worst in terms of healthy food access had a significantly higher obesity incidence and prevalence [41]. One potential interpretation of this finding is that participants perceiving lack of access as a significant problem are expressing barriers constraining their food shopping-related choices, such as the foods purchased, frequency of shopping trips, and transportation to stores. We have previously shown that residents’ perceptions of relatively lower availability of healthful foods and relatively greater lack of access are not particularly correlated with spatial availability measures of the built food environment but are strongly associated with household socioeconomic characteristics, including food insecurity [43].

However, arriving at a causal interpretation from observational research on food shopping characteristics is complicated by the fact that it is entirely possible that people who shop at a particular store type (e.g., supercenter or discount store) may differ systematically from those who shop at different types of stores, including in their individual socioeconomic characteristics but also in attributes of the economic, social, and cultural system in which they reside [44], which in turn could influence the types of foods purchased and eaten, leading to obesity [45]. This phenomenon can manifest as selection bias (e.g., differing sampling fractions of study participants who shop at a certain store type and eat healthful foods). Conversely, it could manifest as an endogeneity problem, either through uncontrolled (i.e., residual) confounding (also known as omitted variable bias) or through reverse causality (i.e., persons with a higher BMI being more likely to choose to shop at supercenters than supermarkets).

Using a unique study design as a selection of shoppers nested within a selection of stores, Chaix et al. (2012) were able to determine that shoppers within the same store type were significantly more similar in terms of socioeconomic characteristics and BMI than shoppers between different store types [11]. Courtemanche and Carden (2011) conducted a contextual study relating the county-level prevalence of Walmart supercenters with individual-level BMI and obesity rates and found after adjusting for endogeneity bias (which they interpret as arising from the non-random nature of Walmart’s locations) using an instrumental variable approach [46] that one additional supercenter per 100,000 residents was associated with an average 0.237 kg/m^2^ higher BMI, which, given their population’s characteristics, equated to half a pound [47]. It is conceivable that given sufficient incentives, individuals could be randomized to utilizing certain grocery stores and followed up over time to observe potential changes in BMI; however, unless adherence to stores is excellent, such a randomized trial could be equally subject to bias.

Several limitations and strengths are worth mentioning. The data presented here are baseline data from a study designed to evaluate a natural experiment. Thus, the study’s size is a function of the recruitment goals, which aimed for a certain sample size and power to detect change over time (and achieved 94% of the enrollment goal of 560 at baseline). Thus, we cannot rule out the possibility that the participants of this study are not representative of all residents of the recruitment area. We can say with certainty, however, that the participants represent a very low-income and largely minority population. This study did not collect data on food prices at all utilized stores, only those at a small subsample of the five most utilized stores, and so we cannot reach any conclusions with respect to an independent role of food prices at the store level and their relationship with BMI. Furthermore, this study lacks data on the types of foods purchased by participants, which is information in an important mediating pathway. Our study focused on BMI as an outcome, instead of overweight/obesity, because 80% of our population was overweight or obese. The key distinguishing feature of our study is that we assessed characteristics of the three most-frequented stores rather than just the single most-frequented store and additionally considered food acquisition at non-retail locations such as food banks/pantries and churches/social service organizations. We also had data available on transportation to the primary store, although we did not find a significant association between this variable and BMI.

## 5. Conclusions

In summary, this study evaluated the association of three distinct sets of food environment-related measures—food shopping and acquisition characteristics, perceptions of the food environment, and GIS-based built environment measures—with BMI levels. Shopping at a supercenter as one’s primary store and reporting that one considered lack of access a significant problem were both associated with higher BMI levels, whereas selecting a smaller store as one’s third most-frequented grocery store was associated with lower BMI levels. We have previously shown in this population that 76% of residents shopped at supermarkets or supercenters for both store 1 and 2 [27].

The positive finding on the supercenter-BMI association raises the questions of whether these types of stores do not have as good or as large a selection of healthy food as supermarkets, if healthier food options within supercenters are placed at a price disadvantage compared to less healthy versions of the same type of food, or if the customers utilizing the stores predominantly make less healthy purchasing decisions. Several studies have shown that supercenters frequently offer among the lowest grocery prices in a given region and thus disproportionately attract customers who are very price conscious [48,49]. Moreover, supercenter utilization was associated with lower educational attainment, lower income, and greater household size in a US telephone survey [45]. In addition, several studies have shown that supermarket utilization was associated with higher income, including in a sample of African-American women from a low-income neighborhood without supermarkets in Detroit [16]. A partial answer to these questions challenging causality could be provided by a study that assesses the nature of purchases and controls for them while evaluating the same research questions we posed here. However, this still leaves open the possibility of residual confounding through foods purchased at other types of food outlets and dietary choices made by individual members of a household and other unmeasured lifestyle factors.

The implications of our study are multi-faceted: First, these findings challenge the concept [50] that limited spatial access to healthy foods, i.e., greater distance to large grocery stores, is an important cause of the obesity epidemic or, conversely, that eliminating food deserts will help curb the obesity epidemic. The vast majority of participants in our study shopped outside of their residential census tract. Food desert residents clearly experience increased grocery shopping burdens, including on average 7.5 extra miles traveled for shopping per week, plus higher fuel and time expenses, as shown in one of our previous studies in South Carolina [10]. However, the present study does not offer evidence that distance is associated with obesity in the population studied here, even though 55% do not travel to their primary store with a car of their own. Second, this study’s findings call into question that all large grocery stores, including supermarkets and supercenters, are beneficial in addressing the food desert and obesity problem. Our study suggests that the food-purchasing behaviors in different types of large grocery store (e.g., supermarkets, supercenters, and warehouse clubs) and motivations and constraints in store selections should be studied in more detail, particularly in under-resourced populations. If future studies find that supercenter customers tend to purchase less nutritious foods, creative new approaches would be needed that combined tailored interventions with system-level incentives toward healthy behaviors, because individually targeted point-of-sales nutrition education programs have not been particularly successful [51].

## Figures and Tables

**Table 1:** Demographic, socioeconomic, and anthropometric characteristics, food shopping and acquisition behaviors, perceptions of the food environment, and built retail environment characteristics of 459 participants from disadvantaged communities in South Carolina (2013–2014).

Characteristics	*n* = 459
**Demographic and socioeconomic characteristics**	
Age (years), mean (SD)	51.9 (14.4)
Women, %	80.0
African American, %	92.6
Married or living together, %	12.9
Adults in household, mean (SD)	1.7 (0.8)
Children in household, mean (SD)	0.6 (1.0)
Education, %	
Less than high school	30.3
High school/GED	38.3
Some college or more education	31.4
Income, %	
$0–9999	46.4
$10,000–19,999	32.9
≥$20,000	20.7
Food security, %	
High or marginal food security	37.7
Low food security	32.7
Very low food security	29.6
Participation in Supplemental Nutrition Assistance Program, %	65.6
Physical activity, number of days/week ≥30 min, mean (SD)	3.2 (2.6)
**Anthropometric characteristics**	
Body mass index, mean (SD)	32.5 (8.9)
Obese, %	54.9
Overweight, %	45.1
**Shopping characteristics of utilized stores**	
**Store 1**	
Distance in miles ^1^, mean (SD)	2.5 (1.3)
Shopping frequency (per week), mean (SD)	1.2 (1.2)
Shopping less than once a week, %	60.4
Store type, %	
Supercenter/warehouse club	27.2
Supermarket	61.2
Other type of store	11.6
Transportation to store 1, %	
Drive own vehicle	45.1
Ride in a friend’s/family member’s car, van etc.	36.0
Take a bus or taxi	8.9
Walk/bicycle	10.0
**Store 2**	
Distance in miles ^1^, mean (SD)	2.7 (1.3)
Shopping frequency (per week), mean (SD)	0.6 (0.6)
Shopping less than twice a month, %	34.4
Store type, %	
Supercenter/warehouse club	22.0
Supermarket	63.6
Other type of store	14.4
**Store 3**	
Distance in miles ^1^, mean (SD)	2.9 (1.3)
Shopping frequency (per week), mean (SD)	0.3 (0.3)
Shopping less than once a month, %	66.0
Store type, %	
Supercenter/warehouse club	13.9
Supermarket	67.1
Other type of store	19.0
**Utilization of community food resources**	
Acquire food at food bank or pantry, %	52.7
Acquire food from church or social service organization, %	53.6
Shop at farmers’ market, %	45.5
**Perceptions of the food environment, mean (SD)**	
Lack of access to adequate food shopping in neighborhood is a problem ^2^	1.5 (1.2)
Availability of healthful foods in neighborhood summed score ^3^	4.7 (3.1)
Many opportunities to purchase fast foods in neighborhood ^4^	1.8 (1.3)
**Built food environment characteristics, mean (SD)**	
Distance to nearest supermarket ^1^, miles	1.5 (0.5)
Distance to nearest supercenter ^1^/warehouse club, miles	2.7 (0.9)
Food desert census tract, %	81.3

^1^ Distances were Winsorized; ^2^ Recoded score ranged from 0 to 3; ^3^ Recoded score ranged from 0 to 12; ^4^ Recoded score ranged from 0 to 4.

**Table 2:** Association of food shopping characteristics, utilization of community food resources, perceptions of the food environment, and built environmental characteristics with body mass index (*n* = 459) in South Carolina (2013–2014).

Characteristics	Model 1			Model 2			Model 3			Squared Semi-Partial Correlation ^1^
	**b**	**SE**	***p***	**b**	**SE**	***p***	**b**	**SE**	***p***	
**Shopping characteristics (utilized grocery stores)**										
**Store 1**										
Distance to store 1 ^2^, miles	0.03	0.43	0.95	0.00	0.44	1.00	0.08	0.45	0.87	0.0001
Store 1 shopping <1 per week vs. ≥1 per week	0.12	0.93	0.90	0.17	0.92	0.85	0.27	0.93	0.77	0.0002
Store 1 type supercenter/warehouse club vs. supermarket	**2.73**	**1.20**	**0.02**	**2.72**	**1.20**	**0.02**	**2.64**	**1.20**	**0.03**	**0.0095**
Store 1 type “other” vs. supermarket	−0.60	1.49	0.69	−0.63	1.49	0.67	−0.62	1.51	0.68	0.0003
Transportation to store 1 with others vs. own vehicle	−1.09	1.04	0.29	−1.38	1.04	0.19	−1.41	1.04	0.18	0.0036
With bus/taxi vs. own vehicle	1.26	1.65	0.45	0.79	1.65	0.63	0.83	1.66	0.62	0.0005
walk/bike vs. own vehicle	−3.13	1.73	0.07	−3.23	1.72	0.06	−3.10	1.73	0.07	0.0063
**Store 2**										
Distance to store 2 ^2^, miles	0.03	0.40	0.95	−0.05	0.40	0.91	−0.01	0.42	0.98	<0.0001
Store 2 shopping <2 times per month vs. ≥2 per month	−1.37	1.05	0.19	−1.43	1.04	0.17	−1.49	1.04	0.15	0.0040
Store 2 type supercenter/warehouse club vs. supermarket	1.79	1.22	0.14	2.04	1.21	0.09	2.06	1.22	0.09	0.0056
Store 2 type “other” vs. supermarket	−0.19	1.29	0.89	−0.02	1.28	0.99	0.05	1.30	0.97	<0.00001
**Store 3**										
Distance to store 3 ^2^, miles	−0.28	0.36	0.44	−0.30	0.36	0.41	−0.31	0.37	0.40	0.0014
Store 3 shopping <1 time per month vs. ≥1 per month	−0.38	1.02	0.71	−0.41	1.01	0.69	−0.49	1.01	0.63	0.0005
Store 3 type supercenter/warehouse club vs. supermarket	1.57	1.33	0.24	1.68	1.33	0.21	1.72	1.34	0.20	0.0032
Store 3 type “other” vs. supermarket	**−2.59**	**1.16**	**0.03**	**−2.60**	**1.15**	**0.02**	**−2.56**	**1.16**	**0.03**	0.0096
**Utilization of community food resources** ^1^										
Acquire food at food bank or pantry vs. not	1.47	0.98	0.14	1.29	0.98	0.19	1.45	0.99	0.14	0.0042
Acquire food from church or other social services vs. not	1.20	0.95	0.20	1.17	0.95	0.22	1.05	0.95	0.27	0.0024
Shop at farmers’ market vs. not	−1.12	0.91	0.22	−1.09	0.91	0.23	−1.12	0.91	0.22	0.0030
**Perceptions of the food environment**										
Lack of access to adequate food shopping in neighborhood ^3^				**−0.87**	**0.39**	**0.03**	**−0.86**	**0.39**	**0.03**	0.0096
Availability of healthful foods in neighborhood summed score ^4^				0.11	0.15	0.45	0.11	0.15	0.49	0.0010
Availability of fast food in neighborhood ^5^				−0.55	0.32	0.09	−0.53	0.33	0.11	0.0050
**Built food environment characteristics**										
Distance to nearest supermarket ^2^, miles							−1.02	1.23	0.41	0.0014
Distance to nearest supercenter or warehouse club ^2^, miles							0.26	0.83	0.75	0.0002
Food desert census tract							1.94	1.57	0.22	0.0030
**Model R-square**	**0.158**			**0.173**			**0.177**			

Adjustment for all models: study location, age, race, gender, marital status, number of adults in household, number of children in household, education, income, food security status, SNAP participation, physical activity. ^1^ Obtained from Model 3; ^2^ Distances were Winsorized; ^3^ Recoded score ranged from 0 to 3; ^4^ Recoded score ranged from 0 to 12; ^5^ Recoded score ranged from 0 to 4. Bold font indicates statistically significant results.
